# Effect of pre-existing psychiatric treatment in suicidal jumpers on the need for in-hospital treatment following injury

**DOI:** 10.1007/s00068-025-02780-3

**Published:** 2025-02-13

**Authors:** Rolle Rantala, Mikko Heinänen, Joonas Kuorikoski, Tuomas Brinck, Tim Söderlund

**Affiliations:** 1 Trauma Unit, HUH Musculoskeletal and Plastic Surgery, Helsinki University Hospital, P.O. Box 266, Helsinki, 00029 HUS Finland; 2 Helsinki University and Helsinki University Hospital, Helsinki, Finland; 3 Department of Orthopaedics and Traumatology, Tampere University Hospital, Tampere, Finland; 4 Department of Orthopaedics and Traumatology, Mehiläinen Hospital, Helsinki, Finland

**Keywords:** Mortality, Trauma, Long-term, Outcome

## Abstract

**Purpose:**

To study the effect of pre-existing psychiatric disorders on outcome following suicidal jump from a height.

**Methods:**

Suicidal jumpers were identified from Helsinki Trauma Registry from 2006 to 2015. Trauma registry data were combined with administrative registry data to obtain long-term mortality and in-hospital treatment. The in-hospital treatment data was from 2 years preceding the index injury and up to 5 years post injury. Reasons for the in-hospital visits were also recorded. We analyzed the patients in two groups, namely patients without a pre-existing psychiatric diagnosis (group 1) and patients with a pre-existing psychiatric diagnosis (group 2).

**Results:**

One-hundred twenty-seven patients were included in the analysis, with 73 patients having received pre-existing psychiatric treatment. A total of 57% of patients were males and 28% of patients suffered severe traumatic brain injury (head AIS ≥ 3). Group 2 patients had a higher number of in-hospital days pre- and post-injury than group 1 patients. Reason for in-hospital treatment in group 2 was psychiatric in over 80% of days except in the year beginning from the index injury. 30-day mortality was similar between the groups 1 and 2 (11% vs. 16%, *p* = 0.395). Five-year survival was 72% in group 2 patients to 86% in group 1 patients (*p* = 0.0001).

**Conclusion:**

Patients with pre-existing psychiatric disorder reaching hospital alive have higher pre- and post-injury requirements for in-hospital treatment than patients without a pre-existing psychiatric disorder. Although pre-existing psychiatric disorder does not affect early mortality, long-term mortality is increased by 14%.

**Trial registration:**

Trial registration number and date of registration: HUS/221/2017, 30.3.2017.

## Introduction

According to the World Health Organization (WHO), roughly 700 000 people commit suicide every year [1]. The method chosen varies widely depending on cultural factors and the environment people live in. Jumping from a height tends to be a more frequent method in densely populated urban areas [2]. In Finland, around 1000 people yearly commit suicide; approximately 6% of women and 4% of men commit suicide by jumping from a height [3]. Most jumpers survive the jump and are treated in hospitals and outpatient clinics thereafter. In a Japanese study, only 15% of the jumpers died at the scene of the jump [4].

A suicidal jump from a height is typically performed at young age and often leads to multiple severe injuries for the survivors [5–12]. Patients suffer from multiple fractures, of which a substantial proportion involves the spinal column, pelvis, and long bones [13–14]. Treatment in primary hospital usually lasts for several weeks and several days are spent in the ICU under mechanical ventilation. Most of these patients (75–88%) require operative treatment as a part of their care [8, 12].

Long-term follow-up studies are missing on use of healthcare resources by suicidal jumpers. One previous study with a 1-year follow-up revealed that only 48% of patients returned to work, one third retired due to disability, and 20% still needed institutional care [5]. Considering these findings and the decreased quality of life among all major trauma patients [15], it is plausible that survivors of a suicidal jump from a height would still have health-related problems and need to contact healthcare services several years after the trauma.

The aim of this study was to investigate the association of suicide attempt by jumping from a height with in-hospital days from 2 years preceding and up to 5 years after trauma, and to evaluate whether a pre-existing psychiatric diagnosis is associated with the need for in-hospital treatment.

## Patients and methods

In southern Finland, the Helsinki University Hospital (HUH) Trauma Unit is responsible for the treatment of adult patients with major trauma. The catchment area of the HUH Trauma Unit during the study period was approximately 1.8 million inhabitants (one third of the Finnish population).

In this study, we included patients treated at the HUH Trauma Unit from 1 January 2006 to 21 December 2015 with (1) New Injury Severity Score (NISS) ≥ 16, (2) age > 16 years, and (3) intentional fall from a height. The Helsinki trauma registry (HTR) was used for patient identification. The HTR includes only patients treated at the HUH Trauma Unit with Injury Severity Score (ISS) ≥ 16 (until 2011) and NISS ≥ 16 (from 2012 onwards). Data quality of the HTR was recently validated and was reported to be excellent [16]. Only the first admission was included in case of several admissions to the HTR for a single patient during the study period. The suicide attempt was recorded in the HTR, and this information was obtained from the medical e-records.

We obtained data on in-hospital days from the care register for social welfare and healthcare (HILMO) maintained by the Finnish institute of health and welfare (THL). It is mandatory for all healthcare providers to register in HILMO the data for every patient with the days spent in hospital combined with diagnosis. The data collected from HILMO can be linked to each person by the Finnish personal identification number. These data were collected from all private and public health care units. Mortality data were obtained from the population registry of Finland.

Only patients who were alive throughout the entire year examined were included for that year’s analysis of the inpatient days. We calculated the percentages of patients with none (0), 1 to 30, and > 30 in-hospital days for the 2 preceding years and up to 5 years from the index injury.

All pre- and post-injury years are calculated for each patient from the date of the index injury, i.e. the first post-injury (Y1) year begins from the date of the index injury and second post-injury year (Y2) begins 365 days after the date of index injury. The in-hospital days reported for the first post-injury year (Y1) include also the acute, initial trauma care after the index injury.

We divided the patients into two groups; group 1 included patients without a pre-existing psychiatric diagnosis (ICD-10 diagnose class) in the 2 years before the index injury and group 2 included patients with a psychiatric diagnosis in the 2 years before the index injury (group 2). Psychiatric diagnoses were retrieved from outpatient and in-hospital data for each patient for 2 years before the index injury.

Severe head injury was defined as head AIS ≥ 3. Expected 30-day mortality was calculated using RISCII [17]. Standard mortality ratio (SMR) (30-day) was calculated using the formula SMR = observed mortality/expected mortality.

Long-term mortality was analyzed using a Kaplan-Meier curve. Controls for each group were identified from a previous study [18]. For each trauma registry patient included in this study, we obtained 10 control persons from national registries by the Population Register Center of Finland. Control persons were matched by age (± 6 months), sex, and county of residency on the day of the accident. By this method we aimed to assess the possible changes in mortality of the study population compared to similar population.

Parameters with normal distribution are presented as mean with standard deviation and parameters with skewed distribution are presented as median with 25% and 75% quartiles. The gathered data were analyzed using SPSS 25.0 and Microsoft Excel. Statistically significant level for *p*-value was set as 0.05. Continuous parameters were analyzed by Mann-Whitney U method. Categorical parameters were compared using Pearson´s chi-square method. R Core Team (2022) R: A language and environment for statistical computing, R Foundation for Statistical Computing, Vienna, Austria (URL https://www.R-project.org/) was used for visualization of graphs.

## Results

### Demographics

During the study period 127 patients fulfilled the inclusion criteria. There were 54 patients (43%) in group 1 and 73 patients (57%) in group 2. Slightly over half of the patients were males (*n* = 73, 58%). Median patient age was 29 years (IQR 21–44 years); only 5 patients (3.9%) were > 65 years old. Patients were severely injured (median NISS 34, IQR 24–48). Median of the first measured GCS (at the scene or in the hospital) was 14 (IQR 12–15). Basic demographics for group 1 and 2 are shown in Table 1.

**Table 1:** Patient demographics

	Group1, *n* = 54	Group 2, *n* = 73	*p*-value
Age, y, mean (± SD)	31 (± 12)	35 (± 16)	0.347
Sex, M:F	36:18	37:36	0.072
Males, %	67	51	
NISS, median (IQ25,75)	34 (24–48)	34 (24–48)	0.754
Admitted to ICU, %	81	86	
ICU days, median (IQ25,75)	6 (2,11)	7 (2,15)	0.461
24-hour mortality, %	9	8	0.837
30-day mortality, %	11	16	0.395
Expected 30-day mortality (RISCII), %	8	13	
SMR	1.32	1.28	
1-year mortality, %	11	19	0.217
Severe Traumatic brain injury, (head AIS ≥ 3), %	31	25	0.395

### In-hospital days

The median and interquartile ranges of in-hospital days in groups 1 and 2 are shown in Table 2. The in-hospital days were constant in the 2 pre-injury years in group (1) In contrast, in-hospital days increased in the year preceding the index injury in group (2) There was also an increase of in-hospital days due to psychiatric reasons in group 2 at the same time.

During the first year after index injury both groups had a higher number of in-hospital days. In group 2, the in-hospital days remained slightly higher than in group 1 for 3 years after the injury (Table 2). The difference was statistically significant. Differences between groups 1 and 2 in the in-hospital days were mostly due to psychiatric reasons (F-days).

**Table 2:** In-hospital days

Year	Y-2	Y-1	Y1	Y2	Y3	Y4	Y5
All days							
Group 1	0 (0, 0)	0 (0, 0)	51 (26, 134)	0 (0, 3)	0 (0, 2)	0 (0, 1)	0 (0, 0)
Group 2	0 (0, 5)	7 (0, 103)	116 (22, 165)	4 (0, 33)	2 (0, 48)	0 (0, 8)	0 (0, 11)
*p*-value	**< 0**,**01**	**< 0**,**01**	**0**,**012**	**0**,**003**	**0**,**014**	0,413	**0**,**021**
F-days							
Group 1	0 (0, 0)	0 (0, 0)	0 (0, 0)	0 (0, 0)	0 (0, 0)	0 (0, 0)	0 (0, 0)
Group 2	0 (0, 5)	5 (0, 33)	6 (0, 59)	0 (0, 27)	0 (0, 14)	0 (0, 7)	0 (0, 10)
*p*-value	**< 0**,**001**	**< 0**,**001**	**< 0**,**001**	**< 0**,**001**	**< 0**,**001**	0,182	**0**,**006**
Non-F-days							
Group 1	0 (0, 0)	0 (0, 0)	53 (26, 132)	0 (0, 3)	0 (0, 3)	0 (0, 0)	0 (0, 0)
Group 2	0 (0, 0)	1 (0, 5)	107 (40, 117)	0 (0, 4)	0 (0, 5)	0 (0, 0)	0 (0, 0)
*p*-value	**0**,**002**	**< 0**,**001**	0,37	0,526	0,569	0,594	0,366

Data are shown as median and 25–75% quartiles. Data were collected only from the patients who were alive the entire year. All, all in-hospital days; F-days, in-hospital days due to psychiatric diagnose; Non-F-days, in-hospital days due to other diagnoses than psychiatric. Statistically significant *p*-values are marked as bold

**Fig. 1:**
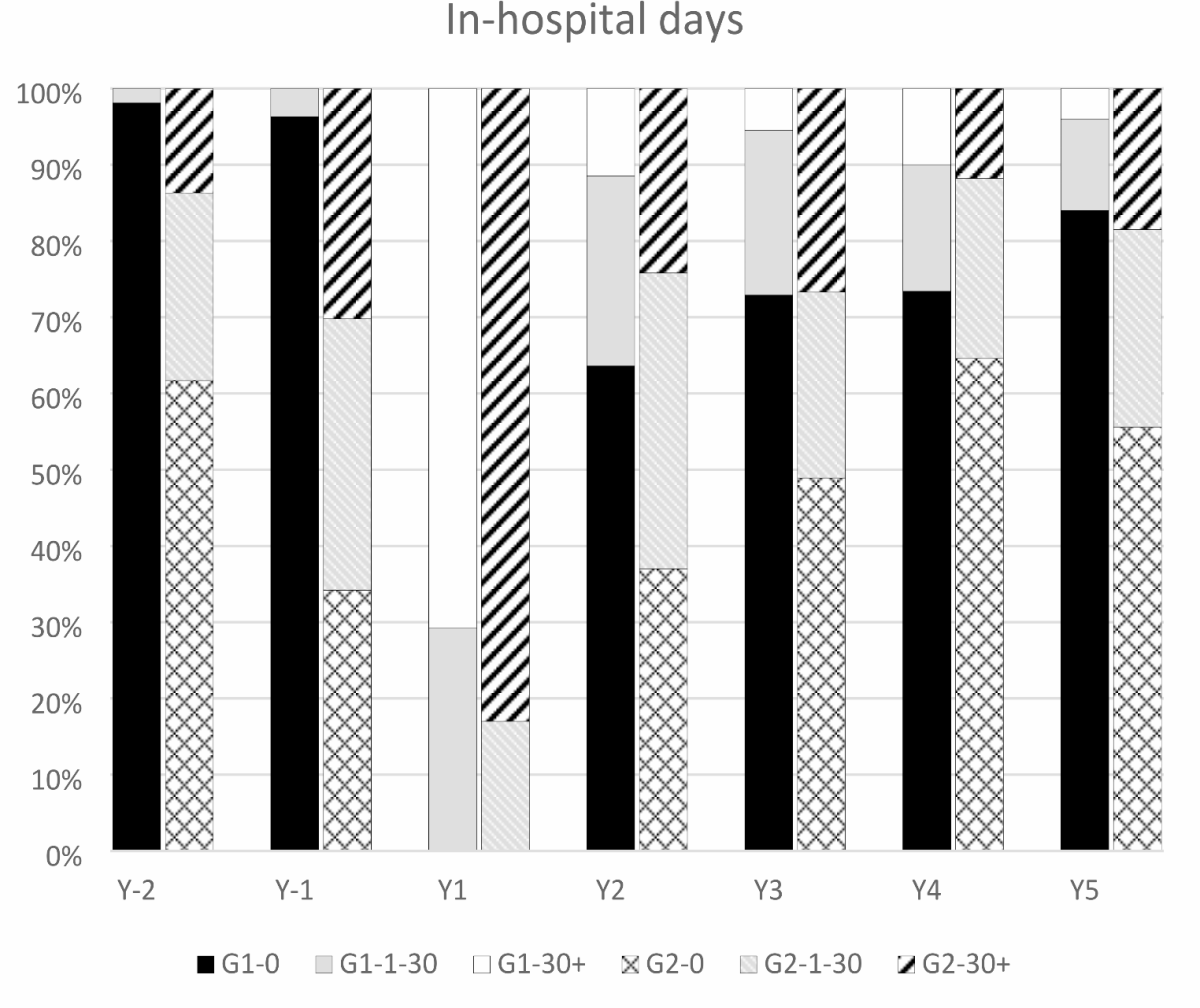
Distribution of in-hospital days due to any diagnosis G1, group 1; G2, group 2; -0, no in-hospital days during the year; -1-30, 1 to 30 in-hospital days during the year; -30+, > 30 in-hospital days during the year

The distribution of in-hospital days within each year was very skewed, with some patients having a high number of days and most patients had none. This was observed in all years except in the year of index injury. The proportion of patients with no in-hospital days, 1–30 in-hospital days, and > 30 in-hospital days for any cause are shown in Fig. 1. For patients without a pre-existing psychiatric diagnosis (group 1) the proportion of patients with no in-hospital days after the index injury did not reach pre-injury levels within 5 years after injury. The proportion of patients with no in-hospital days in group 2 (pre-existing psychiatric diagnosis) was lower for the first 2 years after the injury before reaching the pre-injury level. The number of patients with no in-hospital days was always higher in group 1 than in group 2. The proportion of patients with > 30 in-hospital days per year was higher in group 2.

**Fig. 2:**
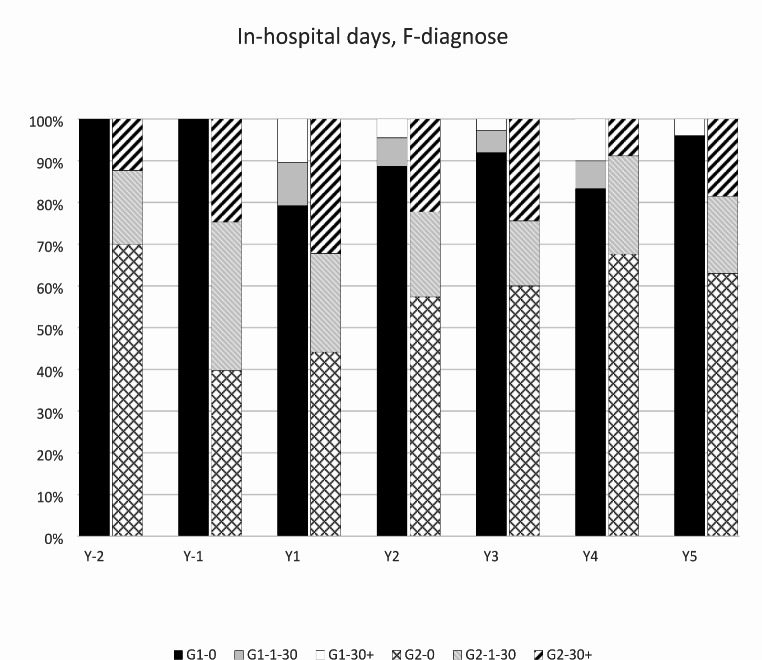
Distribution of in-hospital days due to psychiatric reasons (F-diagnose) G1, group 1; G2, group 2; -0, no in-hospital days during the year; -1-30, 1 to 30 in-hospital days during the year; -30+, > 30 in-hospital days during the year

The distribution of in-hospital days due to a psychiatric cause is shown in Fig. 2. Most in-hospital days were due to psychiatric reasons except in the year after the index injury in group 2.

A comparison on the number of in-hospital treatments due to psychiatric reasons (F-diagnosis) for all reasons is shown in Table 3. In-hospital days in group 2 were mainly due to psychiatric reasons except in the year after the index injury. In group 1, in-hospital days were due to non-psychiatric reasons from pre-injury to 3 years after the index injury. After 3 years post-injury, the in-hospital days were mainly due to psychiatric reasons in both groups.

**Table 3:** Percentages of in-hospital days due to psychiatric diagnoses compared with all diagnoses

	Y-1	Y-2	Y1	Y2	Y3	Y4	Y5
Group 1, %	0	0	9	39	29	92	96
Group 2, %	95	85	33	94	93	96	96

Calculated for patients with at least one in-hospital day per year

### Mortality

**Fig. 3:**
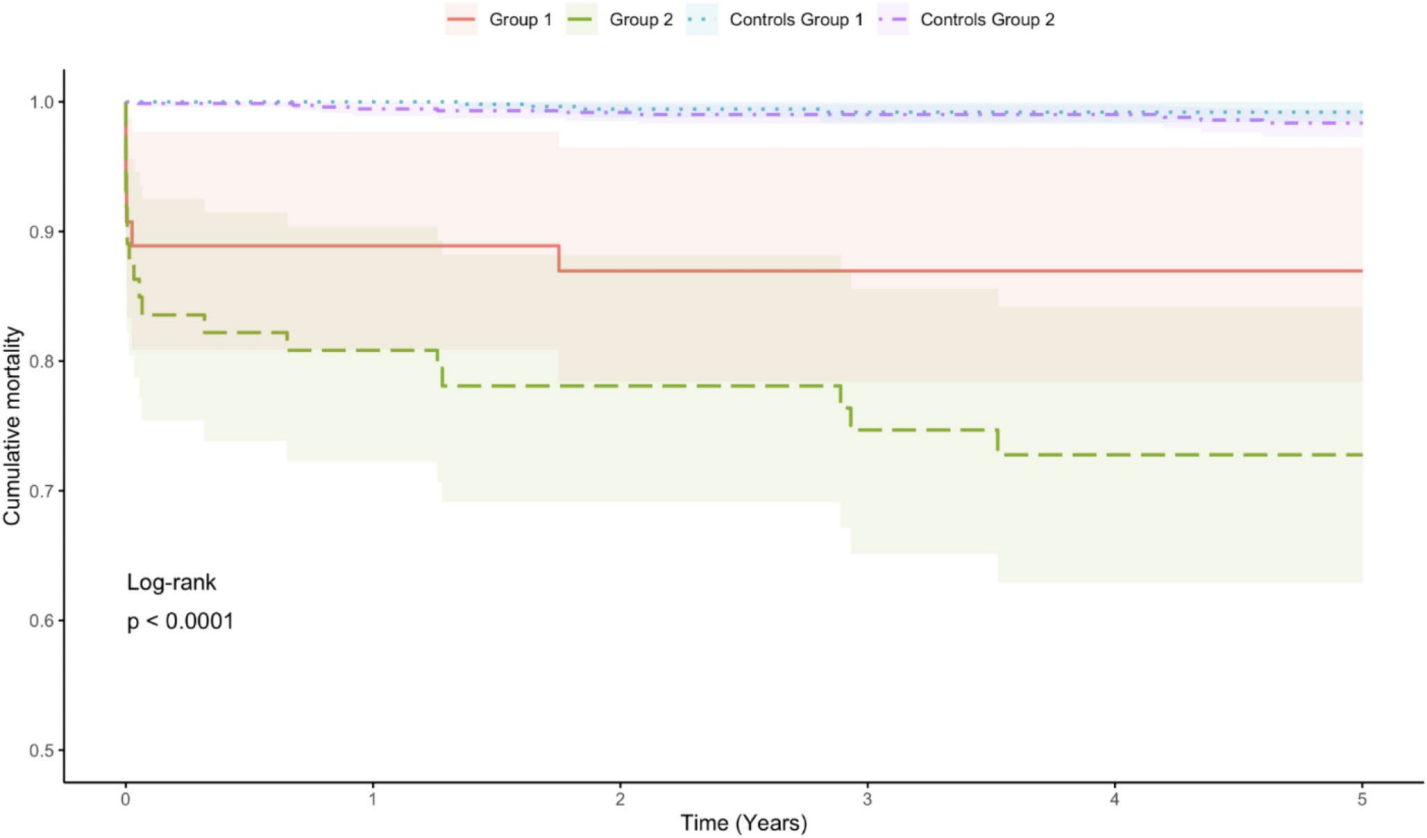
Kaplan-Meier survival analysis No preinjury F dg, group 1; A preinjury F dg, group 2; Control 1, controls for group 1; Control 2, controls for group 2

The 24-hour and 30-day mortalities are shown in Table 1. There was no major difference between the groups. The calculated 30-day SMRs were similar in both groups. The Kaplan-Meier survival up to 5 years is shown in Fig. 3. The overall long-term survival was lower in group 2, with a 14% higher mortality in group 2 than in group 1 at 5 years (*p* = 0.0001).

## Discussion

Our study revealed that among suicidal jumpers who survive the jump, the in-hospital days were higher for several years after the index injury compared with levels preceding the event. The in-hospital days were mainly due to psychiatric reasons. This is a new finding, as no other previously published study has presented long-term follow-up data in this patient group on use of healthcare resources.

A somewhat surprising finding in this study was that the in-hospital days spent began to increase already 1 year before the suicidal jump in patients with a pre-existing psychiatric diagnosis. Plausible explanations may include that the psychiatric or somatic morbidity that eventually leads to the suicide attempt begins to manifest a few years before the jump. This is seen in the results as increased in-hospital days in the year preceding the jump. A previous study revealed that the risk of suicide is higher among cancer patients than in patients with another somatic morbidity [19]. Previous studies have also established that the patient’s risk of suicide is several-fold higher during the first few months following psychiatric inpatient care [20] and this pattern may be observed in our results as an increase in in-hospital treatment in the years preceding the jump. This emphasizes the importance of suicide prevention during and following psychiatric inpatient care. However, the significance of this finding in our study is uncertain due to the relatively short pre-trauma follow-up period of only 2 years. More variation in the parameters could occur with a longer pre-injury follow-up period. The use of healthcare resources in the month preceding death by any suicide method in the elderly population was recently reported [21]. Only 54% of the elderly patients had a healthcare contact in the month preceding death and only 28% had a contact due to psychiatric problems.

The high NISS score indicates that these patients were severely injured. Median age was lower than in the entire blunt trauma population in HTR (29 vs. 53 years) [18]. These findings are consistent with previous studies published on suicidal jumpers. We therefore conclude that suicidal jumpers that survive to hospital are younger than average trauma patients and the in-hospital treatment is demanding for these patients and for the healthcare system [5, 7, 8, 11, 12, 14, 22–26].

Four previous studies on suicidal jumpers only reported in-hospital mortality that varied from 7.5 to 21.4% [7, 8, 11, 12]. These studies were heterogenous, as the total number of patients included ranged from 40 to 1070. Three of the study populations were smaller (40–64 patients) and did not report ISS inclusion criteria [7, 11, 12]. Topp et al. had the largest study population of 1040 suicidal jumpers and included patients with ISS > 9 (mean 31.8) [8]. They reported an in-hospital mortality of 21.4% [8]. In our study, the 30-day mortality was 14.2%. Variability in in-hospital mortality could be explained by the differences between patient demographics, since our study seemed to have significantly younger patients (median age 29 years) when compared with the patients in Topp et al. (mean age 39.6 years) [8]. Trauma patients with pre-existing psychiatric disorder or suicide attempt have higher risk for in-hospital mortality and longer hospital stay [27]. In the current study, the mortality was similar in both groups but the patients with pre-existing psychiatric disorder had higher number of in-hospital days in the first year after the index injury.

Only one previous study on suicidal jumpers only reported longer-term mortality, which was 17.5% at 1 year [12]. The 1-year mortality was 15.7% in this study. The mortality between group 1 and 2 increased in the long-term, with a 14% higher 5-year mortality in patients with a pre-existing psychiatric diagnosis. Our findings on long-term mortality are new, as no other previous study on suicidal jumpers only has presented mortality data up to 5 years. A previous study on patients with major blunt trauma revealed a 5-year mortality of approximately 30% [18], which is somewhat higher than that observed in the present study. However, the patients were significantly older in the previous study; median age was 53 years compared with the median age of 29 years in the current study. The previous study also included isolated head injuries, which probably explains at least some of the difference in mortality rates. Furthermore, only 28% of the population in this study had suffered a severe traumatic brain injury.

In previous publications, a large proportion of jumpers had contact with psychiatric services or had a psychiatric diagnosis in their records before the suicidal jump [5, 6, 23]. This study is consistent with these findings. Almost half of the patients in this study did not have any contact with healthcare professionals before the suicidal jump due to psychiatric reasons. One possible explanation is that for some of these patients, the suicide attempt is the first manifestation of psychotic illness and psychosis [6]. Mood disorders and psychotic illnesses appear to be the most common psychiatric morbidities among the suicidal jumpers [22, 24, 28]. It is possible that in many of these cases, there might have been an opportunity to prevent major catastrophic events, such as deliberate jump from a height, as the healthcare system had a prior contact with these patients. Limited resources and the problem of identifying risk factors for suicidal acts may be the most substantial challenges in preventing these acts. A recent study showed that young adults who had been treated in-hospital for a fracture had a 6-fold higher risk of death in a 10-year follow-up compared with the general population [29]. Suicide (28%) was the leading cause of death for these patients. Fractures requiring in-hospital treatment in young adults may indicate suicidal behavior in later life. One study [30] reported long-term (6 ± 3 years) outcomes on the patients (*n* = 35) with pre-existing psychiatric disorder after suicide attempt by jumping from height or leaping in front of train or car. Good or satisfactory quality of life was observed in half of the patients at the end of the follow-up.

It is currently established that if the psychiatric disorder escalates to the point of suicide attempt, the effects are catastrophic for the patients and relatives and are expensive for healthcare systems. Therefore, it would be reasonable to invest more resources towards prevention, such as identifying these patients. However, since almost half of the patients did not have any pre-existing psychiatric diagnosis before the suicide attempt, other measures are also necessary. Some concrete evidence exists on the measures that can be taken to prevent specifically deliberate jumps from a height; bridges are known destinations of suicidal jumpers, probably due to their popularity and well-known locations [27, 31, 32]. Addition of protective fences to bridges that make access to a jumping location more difficult reduced the amount of suicidal jumps from the bridges in question [28].

### Strengths and limitations

The strengths of this study include the fairly large number of patients from a 12-year time-period, with complete medical records obtained from three different registries. This was also the first study with a 5-year follow-up of healthcare resource utilization after suicidal jumps from a height.

The nature of registry-based research can be considered both a limitation and a strength of our study. The HTR included only patients with NISS ≥ 16, hence suicidal jumpers with more minor injuries were not included in the analysis. However, the trauma unit of the HUH is the only hospital in the catchment area that treats patients with major blunt trauma, and we were able to capture a fairly accurate picture of this patient group as the HTR includes all patients admitted to our hospital within the registry inclusion criteria. The HTR has been routinely maintained for several years and the data quality of the HTR has been considered as excellent and therefore our data can be considered reliable [16].

However, it is possible that the intent behind the patient’s jump from height is not always accurately recorded in the medical e-records due to human errors in data imput or, for example, the patient’s reduced level of consciousness during treatment. Therefore, some cases may be missing from the study.

Regarding patient grouping, it is possible that a patient may have had a pre-injury psychiatric disorder without any visits to healthcare providers in 2 years and therefore was not included in the group 2.

Our study included only patients who attempted suicide by jumping from a height. Therefore, our results cannot be generalized to patients who attempt suicide with other methods.

## Conclusion

Patients with a pre-existing psychiatric disorder reaching hospital alive have a higher pre- and post-injury requirement for in-hospital treatment than patients without a pre-existing psychiatric diagnosis. The pre-existing psychiatric diagnosis did not influence early mortality, but long-term mortality was increased by approximately 14%.

## Data Availability

Data reported is presented in the manuscript.
